# Sphingolipid Expression During Corneal Wound Healing in a Sphingosine Kinase 1 Knockout Model

**DOI:** 10.3390/cells15080733

**Published:** 2026-04-21

**Authors:** Sandip K. Basu, Steve Mabry, Peter Nsiah, Sarah E. Nicholas, Nataliya Lenchik, Mark Altawil, Chi-Yang Chiu, Daniel J. Stephenson, Charles E. Chalfant, Dimitrios Karamichos, Nawajes Mandal

**Affiliations:** 1 Department of Ophthalmology, University of Tennessee Health Science Center, Memphis, TN 38163, USA; sbasu8@uthsc.edu (S.K.B.); nlenchik@uthsc.edu (N.L.); maltawil@uthsc.edu (M.A.); 2 North Texas Eye Research Institute, University of North Texas Health Science Center, Fort Worth, TX 76107, USA; steve.mabry@unthealth.edu (S.M.); peternsiah@my.unthsc.edu (P.N.); sarah.nicholas@unthealth.edu (S.E.N.); 3 Department of Family Medicine, Texas College of Osteopathic Medicine, University of North Texas Health Science Center, Fort Worth, TX 76107, USA; 4 Department of Preventive Medicine, University of Tennessee Health Science Center, Memphis, TN 38163, USA; chiu@uthsc.edu; 5 Departments of Medicine and Cell Biology, University of Virginia School of Medicine, Charlottesville, VA 22903, USA; stephensondj@alumni.vcu.edu (D.J.S.); krj2sf@uvahealth.org (C.E.C.); 6 Research Service, Richmond Veterans Administration Medical Center, Richmond, VA 23298, USA; 7 Department of Anatomy & Neurobiology, University of Tennessee Health Science Center, Memphis, TN 38163, USA; 8 Memphis VA Medical Center, Memphis, TN 38104, USA

**Keywords:** sphingolipids, sphingosine kinase 1, sphingosine-1-phosphate, ceramide, cornea, wound healing

## Abstract

**Highlights:**

**What are the main findings?**

- Wild-type mice exhibit large shifts in corneal sphingolipids during wound healing, particularly within the first two weeks after injury.
- Sphingosine kinase 1 knockout blunts wild-type patterns of corneal sphingolipid expression during wound healing.

**What are the implications of the main findings?**

- Sphingosine kinase 1 and its metabolic product sphingosine-1-phosphate function as modulators of sphingolipid metabolism during corneal wound healing.
- The first two weeks following corneal injury may represent an ideal window of opportunity for therapeutic interventions that target sphingolipids.

**Abstract:**

Corneal scarring is a result of unregulated fibrotic processes in wound healing, which causes visual impairment. Bioactive sphingolipids (SPLs) are known to modulate physiological processes that are central to wound healing. Of these bioactive SPLs, sphingosine-1-phosphate (S1P) is perhaps the most studied. Previous research has shown that knocking out sphingosine kinase 1 (Sphk1), which produces S1P, alters SPL species metabolism and improves wound healing in mice corneas. However, it is unknown how SphK1 knockout (*SphK1^-/-^*) affects SPL metabolism during stages of corneal wound healing. Following an alkali burn procedure on wild-type (WT) and *SphK1^-/-^* mice, corneal lipidomic profiles in unburned corneas at 1, 7, 14, and 28 days post-injury (DPI) were measured. Significant differences in SPL species between genotypes, both in uninjured mouse corneas and during distinct stages of corneal burn healing, were observed. WT mice expressed burn healing stage-dependent modulation of SPL species, with decreased expression of most SPL species observed at 1 and 14 DPI. Interestingly, this wild-type SPL modulation was absent in most measured SPL species in the *SphK1^-/-^* corneas. These findings provide evidence for a previously unknown modulatory role of SphK1 and S1P on the expression of SPLs during corneal wound healing.

## 1. Introduction

The cornea is the eye’s outermost transparent layer, which serves to protect its internal structures from infection and refract light onto the retina. Corneal scarring, due to either injury or disease, is a leading cause of blindness globally [1,2]. Corneal wound healing involves coordinated cascades of cellular events, including cell death, migration, proliferation, differentiation, and extracellular matrix remodeling, all regulated by a complex interplay of signaling molecules [3,4,5,6]. Aberrant wound healing results in fibrotic corneal scar formation through over-accumulation of a disorganized extracellular matrix [4,5,6,7]. While there are topical pharmaceuticals that can decrease the development of these fibrotic scars, therapies to prevent scarring remain elusive, and reversing scarring is an even greater challenge [5,6]. In severe cases, surgical interventions such as keratoplasty or corneal transplantation may be necessary to restore sight [5,6]. Therefore, understanding the molecular mechanisms underlying proper corneal wound healing is critical for the development of novel treatments to prevent fibrotic scar formation and maintain clear vision.

Sphingolipids (SPLs) are crucial components of the plasma membrane in numerous cell types [8,9]. Bioactive SPLs play a major role in cell signaling and are key factors in various physiological processes, including cell migration, survival, contraction, proliferation, gene expression, and cell–cell interactions [10,11,12,13,14]. One critical bioactive SPL that has been extensively studied is sphingosine 1 phosphate (S1P). S1P is synthesized by the phosphorylation of sphingosine (Sph) by either sphingosine kinase 1 (SphK1) or sphingosine kinase 2 (SphK2) [8,9]. There is mounting evidence that S1P signaling may modulate disease pathology in conditions such as atherosclerosis, diabetes, cancer, and inflammatory disorders [15,16,17,18]. S1P signaling has been shown to modulate various wound healing mechanisms, including angiogenesis, immune cell infiltration, and mitochondrial function [14,19,20,21]. S1P, SphK1, S1P receptors, and all proteins involved in S1P signaling can be collectively referred to as the SphK1-S1P axis [21,22,23]. Many groups have pharmacologically inhibited the SphK1-S1P axis and observed improved wound healing outcomes [24,25,26].

In the cornea, very few studies have been conducted on S1P activity; however, the role of S1P signaling in other ocular tissues, such as the retina, RPE-choroid, and trabecular meshwork, has been shown to be essential for cellular adhesion, inflammation, and neovascularization [27,28,29,30]. Our group has demonstrated that the SphK1-S1P axis is a critical component of corneal wound healing and fibrosis both in vitro and in vivo. Downregulation of both SphK1 expression and activity resulted in anti-fibrotic effects in human corneal fibroblasts [31]. S1P signaling also regulates multiple signaling proteins in transforming growth factor β (TGF-β)-mediated corneal fibrosis [32,33]. We have further shown that corneal neovascularization is reduced in SphK1 knockout (*SphK1^-/-^*) mice during the wound healing process [34]. However, the effects of SphK1 knockout on SPL concentrations during corneal wound healing have not yet been fully elucidated. To determine how SPL species concentrations shift during wound healing, we performed targeted lipidomics analysis. We performed analysis of specific SPL subspecies within the corneas of wild-type (WT) and *SphK1^-/-^* mice at 1, 7, 14, and 28 days post-injury (DPI). We sought to discover genotype-specific patterns of SPL concentrations over time, with the goal of identifying critical timepoints in wound healing for therapeutic intervention. Time-dependent patterns of SPL concentrations could be responsible for improved wound healing, which we have previously reported [34].

## 2. Materials and Methods

### 2.1. Animal Care

In this study, 10–15-week-old *SphK1^-/-^* mice (gift from Dr. Richard L. Proia, NIDDK, Bethesda, MD) in an albino (BALB/c) background were utilized, and their littermate wild-type BALB/c (WT) were used as controls. All the mice were born and raised in the University of Tennessee Health Science Center (UTHSC) vivarium, following its guidelines for animal housing. Tail clippings were collected during the weaning process and were used for genotyping to identify the WT and *SphK1^-/-^* littermates. All mice were maintained in dim (5–10 lux) cyclic light (12 h. ON/OFF) from birth. All procedures were performed according to the Association for Research in Vision and Ophthalmology Statement for the Use of Animals in Ophthalmic and Vision Research and the UTHSC Guidelines for Animals in Research. All animal studies were reviewed and approved by the UTHSC Institutional Animal Care and Use Committee (IACUC).

### 2.2. Corneal Injury by Alkali Burn

Corneal injury by alkali burn was performed bilaterally following previously published procedures [34]. Briefly, before the procedure, the mice were anesthetized with an intraperitoneal injection of ketamine (100 mg/kg body weight; Henry Schein, Melville, NY, USA) and xylazine (5 mg/kg body weight; Henry Schein, Melville, NY, USA). A topical application of 0.5% proparacaine hydrochloride (Alcon Laboratories, Fort Worth, TX, USA) was applied as a pre-injury analgesic to the corneal surface of each eye. Using a dissection microscope, a 2 mm round piece of Whatman No. 1 filter paper, soaked in 0.5 N NaOH, was then applied to the central cornea of one eye for 20 s. After 20 s, the eye was immediately rinsed with 0.9% sterile saline solution for approximately 20 s. Following the injury procedure, a topical antibiotic, Erythromycin Ophthalmic Ointment 0.5% (Bausch & Lomb, Rochester, NY, USA), was applied to the burned cornea. This procedure was then repeated on the opposite eye. Subcutaneous injection of Buprenorphine SR (0.6 µg/kg body weight; ZooPharm, Laramie, WY, USA) was used as a post-injury analgesic. The mice were kept on warm pads and monitored to ensure that they woke from the anesthesia and were returned to their original housing.

### 2.3. Mass Spectrometry Analysis of Sphingolipids

For SPL analysis, corneas were harvested from both WT and *SphK1^-/-^* mice at the specified DPI by dissecting the corneas along the limbus line, carefully excluding the limbal vessels. Four corneas, isolated from two individual mice, were used to constitute one “*n*”, with *n* = 6 for each genotype at each timepoint. Sample size was determined from a power analysis using our previously published experiments [34]. Following isolation, corneas were weighed and stored at −80 °C after freezing in liquid nitrogen. The corneal sphingolipid extraction and mass spectrometric analysis were performed from each “*n*” (four corneas in total) at the University of Virginia, Charlottesville, VA, USA, following previously published protocols [35]. The individual sphingolipid species were identified and quantified in a semi-quantitative manner from peak areas relative to internal standards. The resulting values were reported in pmol/mg of tissue to standardize the data for comparison. If undetectable, the values were reported as either zero or “ND.” The detection cutoffs used for Cer, SM, and MHC were >100 signal-to-noise ratio (S/N) and >10 S/N ratio for the low-expressing species like SA, Sph, and S1P. The method used for sphingolipid analysis measures the non-dihydro species of Cer, MHC, and SM, but not other lipid classes like wax esters, cholesterol esters, or phospholipids beyond SM [36].

### 2.4. Statistical Analysis

Statistical analyses for overall SPL concentrations and major SPL family concentrations were performed using GraphPad Prism 10 (Version 10.6.1, San Diego, CA, USA). Normal data distribution was verified using the Shapiro–Wilk test. To determine whether genotype and timepoint interacted in major SPL family concentrations, 2-way repeated measures ANOVA followed by Fisher’s LSD for post hoc analysis was performed. To identify which individual SPL species were driving these family-wide effects, we conducted additional analyses on each SPL analyte. Within-genotype comparisons were conducted using paired *t*-tests to assess differences between each follow-up timepoint (days 1, 7, 14, and 28) and baseline (unburned). Between-genotype comparisons were evaluated using two-sample *t*-tests at each timepoint. All tests were two-sided, and *p*-values were adjusted for multiple comparisons using the Benjamini–Hochberg procedure to control the false discovery rate. Analyses were performed in R (version 4.5.0). Significance was defined as *p* < 0.05. All results are reported as mean ± S.E.M unless otherwise noted. All results were graphed using GraphPad Prism.

## 3. Results

### 3.1. Corneal SPL Profiles of WT and SphK1^-/-^ Mice

**Figure 1:**
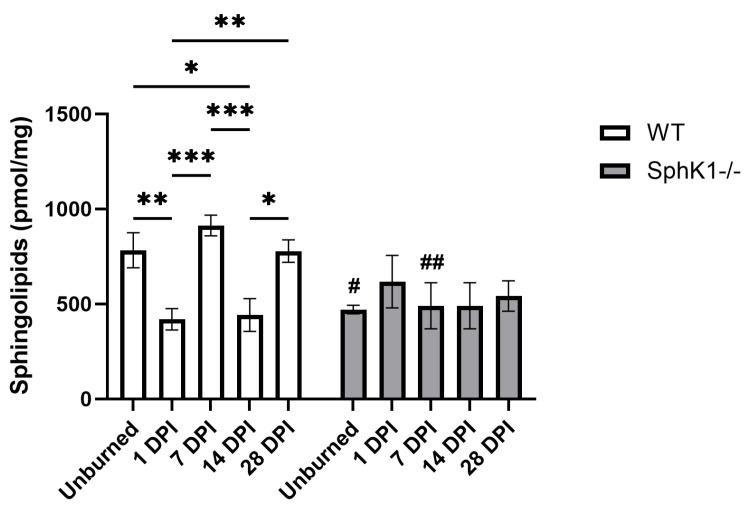
Sphingolipid metabolism is blunted by sphingosine kinase 1 knockout. All data are represented as mean ± S.E.M. Statistical significance was evaluated by two-way repeated measures ANOVA with Fisher’s LSD. Significant differences within genotype are indicated as * *p* < 0.05; ** *p* < 0.01; *** *p* < 0.001. Significant differences between genotypes are indicated as # *p* < 0.05; ## *p* < 0.01. *n* = 6 for all groups. DPI: days post-injury; *SphK1^-/-^*: sphingosine kinase 1 knockout; WT: wild-type.

We observed a main effect of genotype (*p* = 0.0221; Figure 1) and an interaction between time and genotype (*p* = 0.0079; Figure 1) in overall SPL concentrations. Specifically, in WT mice corneas, overall SPL concentrations decreased compared to unburned corneas after 1 DPI (*p* = 0.0076; Figure 1) and 14 DPI (*p* = 0.0119; Figure 1) and were not different at 7 or 28 DPI (Figure 1). WT SPL concentrations at both 1 DPI and 14 DPI were significantly decreased compared to 7 DPI (1 DPI *p* = 0.0005; 14 DPI *p* = 0.0008; Figure 1) and 28 DPI (1 DPI *p* = 0.0083; 14 DPI *p* = 0.0130; Figure 1). Conversely, overall SPL concentrations in *SphK1^-/-^* mice did not change, regardless of time post-corneal burn (Figure 1). *SphK1^-/-^* corneas, both unburned (*p* = 0.0179; Figure 1) and 7 DPI (*p* = 0.0017; Figure 1), were decreased compared to WT corneas at the same timepoint. We have further separated the SPLs by major families to determine if these patterns are reflected by the SPL families, or within individual SPL species.

### 3.2. Ceramides

**Figure 2:**
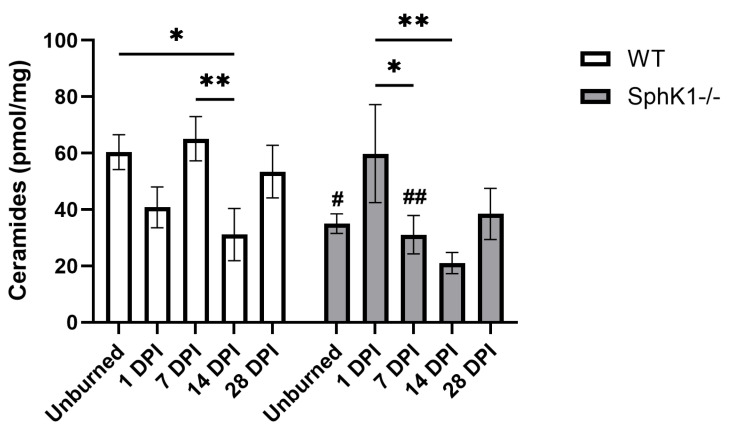
SphK1 knockout induced limited blunting of ceramide expression during wound healing in mouse corneas. All data are represented as mean ± S.E.M. Statistical significance was evaluated by two-way repeated measures ANOVA with Fisher’s LSD. Significant differences within genotype are indicated as * *p* < 0.05; ** *p* < 0.01. Significant differences between genotypes are indicated as # *p* < 0.05; ## *p* < 0.01. *n* = 6 for all groups. DPI: days post-injury; *SphK1^-/-^*: Sphingosine kinase 1 knockout; WT: wild-type.

We observed a main effect of genotype (*p* = 0.0436; Figure 2) and an interaction between time and genotype (*p* = 0.0478; Figure 2) in total Cer concentrations. Cer concentrations from WT mouse corneas decreased at 14 DPI compared to unburned corneas (*p* = 0.0237; Figure 2). WT corneal Cer concentrations were also decreased at 14 DPI compared to 7 DPI (*p* = 0.0093; Figure 2). Interestingly, Cer concentrations were also decreased in *SphK1^-/-^* mouse corneas, but only at 7 DPI (*p* = 0.0262; Figure 2) and 14 DPI (*p* = 0.0034; Figure 2) compared to 1 DPI. Mirroring the pattern observed in overall SPL concentrations, unburned (*p* = 0.0473; Figure 2) and 7 DPI (*p* = 0.0089; Figure 2) Cer concentrations of *SphK1^-/-^* corneas were decreased compared to WT corneas at the same timepoint.

Table 1 summarizes the major species of Cer from both WT and *SphK1^-/-^* mouse corneas, unburned and at each timepoint DPI (Figure S1). Many Cer species decreased at 1 DPI in WT mice (Table 1), including C18:0 (*p* < 0.05), C18:1 (*p* < 0.05), C20:0 (*p* < 0.05), C24:0 (*p* < 0.05), and C24:1 (*p* < 0.05). Fewer Cer species decreased in WT mice at 14 DPI (Table 1), as only C20:0 (*p* < 0.05) and C24:0 (*p* < 0.05) were different from unburned corneas at 14 DPI. Many Cer species were decreased in unburned *SphK1^-/-^* mouse corneas when compared to WT (Table 1), including C16:0 (*p* < 0.05), C18:0 (*p* < 0.05), C18:1 (*p* < 0.05), C20:0 (*p* < 0.01), C22:0 (*p* < 0.05), C24:1 (*p* < 0.05), C26:0 (*p* < 0.01), and C26:1 (*p* < 0.01). All Cer species decreased in *SphK1^-/-^* at 7 DPI when compared to WT (Table 1). In contrast to the WT, we observed no differences in Cer species in *SphK1^-/-^* between unburned corneas at 1 DPI (Table 1). At 14 DPI, C24:0 (*p* < 0.05), C26:0 (*p* < 0.001), and C26:1 (*p* < 0.05) decreased in *SphK1^-/-^* compared to unburned corneas.

### 3.3. Monohexosyl Ceramides

**Figure 3:**
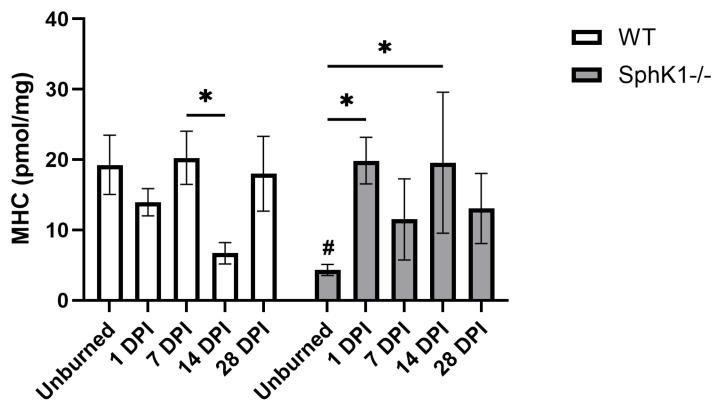
MHC expression decreased at baseline in *SphK1^-/-^* corneas compared to WT. All data are represented as mean ± S.E.M. Statistical significance was evaluated by two-way repeated measures ANOVA with Fisher’s LSD. Significant differences within genotype are indicated as * *p* < 0.05. Significant differences between genotypes are indicated as # *p* < 0.05. *n* = 6 for all groups. DPI: days post-injury; MHC: monohexosylceramide; *SphK1^-/-^*: sphingosine kinase 1 knockout; WT: wild-type.

We observed a significant interaction between genotype and time in MHC concentrations (*p* = 0.0247; Figure 3). In WT mouse corneas, a decrease in MHC concentration at 14 DPI compared to 7 DPI was observed (*p* = 0.0388; Figure 3). Conversely, overall MHC concentration was increased in *SphK1^-/-^* corneas at 1 DPI (*p* = 0.0190; Figure 3) and 14 DPI (*p* = 0.0211; Figure 3) compared to unburned corneas. MHC concentrations were only decreased in *SphK1^-/-^* corneas compared to WT corneas when unburned (*p* = 0.0346; Figure 3).

Table 2 summarizes the major species of MHC from both WT and *SphK1^-/-^* mouse corneas, unburned, and at each timepoint DPI (Figure S2). In WT mice, some of the MHC species were significantly different at 1 DPI compared to unburned corneas (Table 2). C16:0 (*p* < 0.05), C16 DH (*p* < 0.05), and C26:0 (*p* < 0.05) increased, whereas C20:0 (*p* < 0.05) decreased. Significant decreases in MHC species were observed at 14 DPI compared to unburned in WT mouse corneas (Table 2), including C20:0 (*p* < 0.05), C22:0 (*p* < 0.05), C24:0 (*p* < 0.05), C26:0 (*p* < 0.01), and C26:1 (*p* < 0.05). When unburned, all MHC species apart from C14:0 were significantly lower in *SphK1^-/-^* corneas compared to WT corneas (Table 2). We also observed that all MHC species except C20:0 were significantly increased at 1 DPI in *SphK1^-/-^* corneas (Table 2). This increase in MHC concentration in *SphK1^-/-^* corneas was not observed at 14 DPI (Table 2), as only C14:0 (*p* < 0.05) and C18:1 (*p* < 0.05) increased compared to unburned corneas.

### 3.4. Sphingomyelins

**Figure 4:**
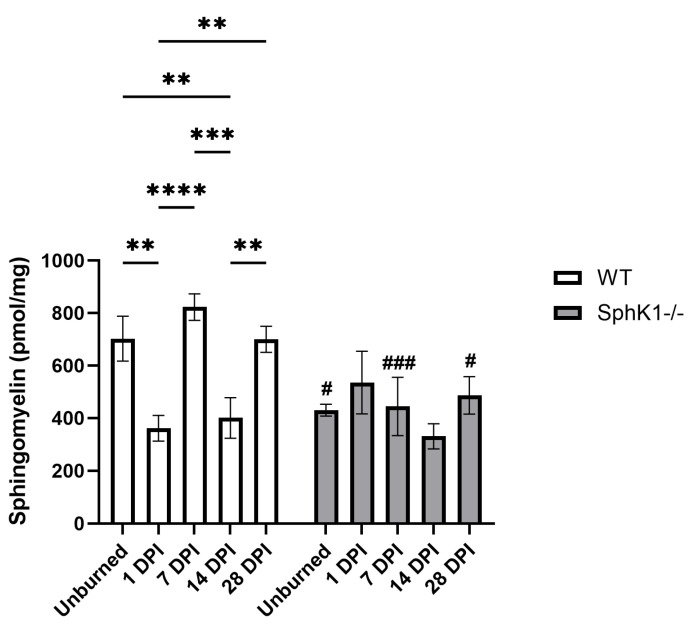
Genotype-specific blunting of sphingomyelin expression observed at baseline, 7, and 28 DPI. All data are represented as mean ± S.E.M. Statistical significance is evaluated by two-way repeated measures ANOVA with Fisher’s LSD. Significant differences within genotype are indicated as ** *p* < 0.01; *** *p* < 0.001; **** *p* < 0.0001. Significant differences between genotypes are indicated as # *p* < 0.05; ### *p* < 0.001. *n* = 6 for all groups. DPI: days post-injury; *SphK1^-/-^*: sphingosine kinase 1 knockout; WT: wild-type.

We observed significant main effects of genotype (*p* = 0.0077; Figure 4), time (*p* = 0.0046; Figure 4), and a significant interaction between genotype and time (*p* = 0.0070; Figure 4) in SM concentrations. SM concentrations were decreased in WT mouse corneas at 1 DPI (*p* = 0.0025; Figure 4) and 14 DPI (*p* = 0.0067; Figure 4) compared to unburned corneas. WT corneal SM concentrations at both 1 DPI and 14 DPI were significantly decreased compared to 7 DPI (1 DPI *p* < 0.0001; 14 DPI *p* = 0.0003; Figure 4) and 28 DPI (1 DPI *p* = 0.0026; 14 DPI *p* = 0.0071; Figure 4). Conversely, SM concentrations did not change over time in *SphK1^-/-^* corneas (Figure 4). As observed in both overall SPL concentrations and Cer concentrations, unburned (*p* = 0.0123; Figure 4) and 7 DPI (*p* = 0.0007; Figure 4) SM concentrations of *SphK1^-/-^* corneas were lower compared to WT corneas. Interestingly, we also observed decreased SM concentrations at 28 DPI compared to WT corneas at the same timepoint (*p* = 0.0472; Figure 4).

Table 3 summarizes the major species of SM from both WT and *SphK1^-/-^* mouse corneas, unburned and at each timepoint DPI (Figure S3). In WT mouse corneas, many SM species were decreased at 1 DPI (Table 3), including C14:0 (*p* < 0.05), C18:0 (*p* < 0.05), C18:1 (*p* < 0.05), C20:0 (*p* < 0.05), C22:0 (*p* < 0.05), C24:0 (*p* < 0.05), C24:1 (*p* < 0.05), and C26:0 (*p* < 0.05). Some SM species also decreased in WT corneas at 14 DPI (Table 3), such as C20:0 (*p* < 0.05), C22:0 (*p* < 0.05), C24:0 (*p* < 0.05), and C26:0 (*p* < 0.05). In unburned mouse corneas, we observed that there were multiple SM species decreased in *SphK1^-/-^* compared to WT (Table 3), which included C16 DH (*p* < 0.05), C20:0 (*p* < 0.05), C22:0 (*p* < 0.05), C24:0 (*p* < 0.05), C24:1 (*p* < 0.05), C26:0 (*p* < 0.05), and C26:1 (*p* < 0.05). Much like the ceramides, all SM species were decreased in *SphK1^-/-^* corneas compared to WT corneas at 7 DPI (Table 3). We observed no changes in SM species at 1 DPI in the *SphK1^-/-^* corneas (Table 3). However, some SM species in *SphK1^-/-^* corneas were decreased at 14 DPI (Table 3), including C16 DH (*p* < 0.05), C24:0 (*p* < 0.01), and C24:1 (*p* < 0.01).

### 3.5. C1P, S1P, and Sphingoid Lipids

**Figure 5:**
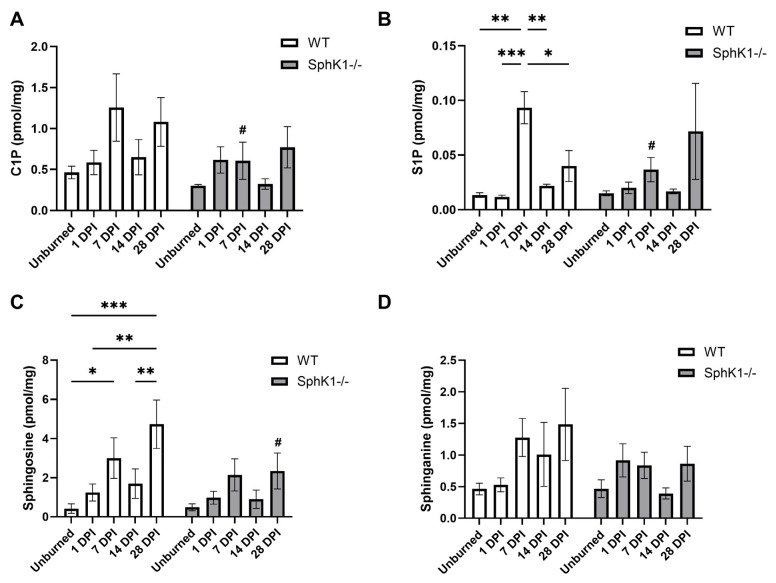
Expression of (**A**) C1P, (**B**) S1P, and the sphingoid lipids (**C**) sphingosine and (**D**) sphinganine in WT and *SphK1^-/-^* mouse cornea after alkali burn. All data are represented as mean ± S.E.M. Statistical significance was evaluated by two-way repeated measures ANOVA with Fisher’s LSD. Significant differences within genotype are indicated as * *p* < 0.05; ** *p* < 0.01; *** *p* < 0.001. Significant differences between genotypes are indicated as # *p* < 0.05. *n* = 6 for all groups. C1P: ceramide-1-phosphate; DPI: days post-injury; S1P: sphingosine-1-phosphate; *SphK1^-/-^*: sphingosine kinase 1 knockout; WT: wild-type.

C1P concentrations did not change over time in WT or *SphK1^-/-^* mouse corneas, regardless of DPI (Figure 5A). However, we observed a main effect of genotypes in C1P concentrations (*p* = 0.0142; Figure 5A). This was primarily driven by decreased C1P concentrations in *SphK1^-/-^* mouse corneas at 7 DPI compared to WT corneas (*p* = 0.0405; Figure 5A). Table 4 summarizes the major species of C1P from both WT and *SphK1^-/-^* mouse corneas, unburned and at each timepoint post-burn. We observed no changes in C1P species post-burn in WT or *SphK1^-/-^* corneas (Table 4; Figure S4). The only C1P species that decreased in *SphK1^-/-^* corneas compared to WT corneas was C14:0 (*p* < 0.05; Table 4).

We observed a main effect of time in S1P concentrations (*p* = 0.0040; Figure 5B). S1P concentrations were increased in WT mouse corneas at 7 DPI compared to unburned corneas (*p* = 0.0012; Figure 5B) and corneas at 1 DPI (*p* = 0.0010; Figure 5B), 14 DPI (*p* = 0.0032; Figure 5B), and 28 DPI (*p* = 0.0249; Figure 5B). S1P concentrations did not change post-burn in *SphK1^-/-^* mouse corneas (Figure 5B). S1P concentrations were increased at 7 DPI in WT corneas compared to *SphK1^-/-^* corneas (*p* = 0.0148; Figure 5B). Much like S1P concentrations, we observed a significant effect of time in Sph concentrations (*p* = 0.0013; Figure 5C). Sph concentrations were increased in WT mouse corneas at 7 DPI compared to unburned corneas (*p* = 0.0194; Figure 5C). Sph concentrations were also increased at 28 DPI compared to unburned (*p* = 0.0002; Figure 5C), 1 DPI (*p* = 0.0022; Figure 5C), and 14 DPI (*p* = 0.0069; Figure 5C). Sph concentrations did not change post-burn in *SphK1^-/-^* mouse corneas (Figure 5C). However, we observed decreased Sph concentrations at 28 DPI in *SphK1^-/-^* mouse corneas compared to WT corneas (*p* = 0.0245; Figure 5C). SA concentration increased in WT mouse corneas at 7 DPI compared to unburned corneas (*p* = 0.0414; Figure 5D). SA concentration did not change post-burn in *SphK1^-/-^* mouse corneas (Figure 5D).

## 4. Discussion

During corneal wound healing, a complex orchestra of cellular events occurs that must be precisely choreographed. In superficial wounds, the epithelial layer of the cornea has sufficient regenerative capacity to prevent serious damage to the central stroma [4,5,6]. In deeper wounds resulting from trauma or disease, resident stromal keratocytes become activated into fibroblasts [3,4,5,6]. Wound healing in these deeper wounds has three general phases: inflammation, proliferation, and remodeling. The initial inflammatory phase is marked by activity of growth factors, such as TGF-β, platelet-derived growth factor (PDGF), and inflammatory cytokines, which stimulate fibroblast activity [4,5,6]. This phase resolves rapidly, typically within 3–5 days [4,5,6]. The proliferative phase occurs simultaneously and extends into the weeks beyond the resolution of inflammation [4,5,6]. During the proliferative phase, corneal keratocytes migrate to the wound site while fibroblasts produce extracellular matrix (ECM) proteins to serve as a cellular scaffold [4,5,6,7]. Concurrently, fibroblasts are activated into myofibroblasts by TGF-β, PDGF, cytokines, and mechanical stress [3,4,5,6,7]. Within approximately 3 weeks, the proliferative phase of wound healing typically transitions into the remodeling phase [4,5,6,7]. However, prolonged activation of myofibroblasts can lead to the development of a corneal scar, which is resistant to tissue remodeling [4,5,6,7]. Though this fibrotic response in many tissues would not disrupt function, scarring in the cornea causes opacity, which limits the visual acuity of patients. Given their modulatory roles in cell proliferation and survival, inflammation, immune cell infiltration, and mitochondrial function, SPLs are central to the wound healing process [8,9,14].

Our study is the first to investigate the corneal SPL profiles of both WT and *SphK1^-/-^* mice during wound healing. Previously, we observed reduced corneal neovascularization in *SphK1^-/-^* mice compared to WT following alkali burn [34]. Recent unpublished data from our group also suggests that *SphK1^-/-^* mice show accelerated wound healing both in corneal epithelium and stroma than WT littermates following alkali burns The concentration of NaOH used in these procedures (0.5 N) causes substantial damage to the corneal epithelium, with minimal damage to the corneal stroma. However, 0.5 N NaOH burns leave sufficient intact tissue for us to conduct biochemical analysis, unlike burns caused by 1.0 N NaOH, which obliterate the entire corneal epithelium [37]. The inclusion of all corneal layers (including the epithelium, stroma, and endothelium) in our lipidomic analysis allows us to present a holistic overview of SPL metabolism in the cornea during wound healing. Alternatively, our ability to interpret our results may be limited by the inclusion of the entire cornea. Since the SPL metabolism of each corneal layer may differ throughout the wound healing process, it is difficult to comment on which cell type contributes most to the changes observed in these SPL profiles.

We observed genotype-specific patterns of SPL concentrations, with lower concentrations of most SPL species observed in *SphK1^-/-^* mice. Both SphK1 knockout and SphK1 silencing have been reported to alter SPL expression in many tissues and disease conditions. For example, Gorshkova et al. found that plasma Cer concentrations increased threefold in *SphK1^-/-^* mice, whereas cardiac Cer levels significantly decreased compared to WT mice [38]. This plasma Cer increase was specifically associated with elevated d22:0-Cer alongside reduced d16:0-Cer [38]. In a study on head and neck squamous cell carcinoma, Shirai et al. observed reduced blood S1P, Sph, and C16:0-Cer concentrations in *SphK1^-/-^* compared to WT mice [39]. However, no difference in total Cer concentrations in blood was observed [39]. SphK1 silencing also promoted cellular senescence and increased Cer and Sph levels in human adipose-derived stromal cells [40]. From these studies and ours, SphK1 knockout and inhibition alter SPL expression in a manner that is highly tissue-specific [8,9].

Inhibiting S1P production via SphK1 knockout shifted SPL metabolism in the cornea, consistent with our previous study [34]. SPL metabolism is centered around Cer, which serves as the precursor for metabolic products that include C1P, SM, MHC, Sph, and other complex SPLs [8,9]. In addition to *de novo synthesis*, C1P, SM, and Sph can be catabolized into Cer [8,9]. SphK1 and SphK2 are localized to different subcellular compartments, where they catalyze the formation of distinct pools of S1P that mediate different functions [22,41,42]. SphK1, localized in the cytosol, generates S1P that mediates cell surface receptor signaling by binding to one of the five S1P receptors (S1PR1–5) [21,22,23]. Conversely, SphK2, located in the nucleus, catalyzes the formation of S1P, which regulates gene expression [21,42]. S1P will either be converted into Sph via S1P phosphatase or irreversibly exit SPL metabolism via S1P lyase [8,9]. Phosphorylation of Sph via SphK1 and SphK2 results in S1P. SphK1 knockdown in vitro increases expression of Cer, SM, SA, and Sph [40]. This effect was reversed using both exogenous S1P and by inhibiting Cer synthase, indicating that down-regulation of SphK1 alters SPL metabolism [40]. As *SphK1^-/-^* inhibits a primary metabolic exit for SPLs, increased recycling of “trapped” SPLs into Cer, MHC, and SM during wound healing may be necessary to support lipid homeostasis [9,40].

Bioactive SPLs have been reported to coordinate wound state-specific molecular mechanisms during tissue repair. In burns, Cer has been reported to stimulate the pro-inflammatory cytokines tumor necrosis factor alpha (TNF-α) and interleukin 1 beta (IL-1β) through activation of nuclear factor kappa B (NF-κB) [43]. SM has been observed to inhibit interleukin 6 (IL-6) signaling in skin, with decreased SM expression increasing the inflammatory response to injury or disease [44,45,46]. Importantly, we observed time-dependent changes in SPL concentrations during wound healing. During wound healing, WT mice exhibited decreased concentrations of major SPL species of Cer, MHC, and SM at 1 and 14 DPI, which were absent in *SphK1^-/-^* mice. Changes in SPL species at 1 DPI coincide with the initial inflammatory phase of wound healing [4,5,6]. Given the reported immunomodulatory effects of SPL species, this may indicate that these decreases in SPL expression are modulating inflammation in the wound [43,44,45,46]. This could further explain the dramatic rebounding of SPL expression observed at 7 DPI in WT mice, as inflammation should be resolved within 5 days [4,5,6]. As we observed decreased SPL concentrations at 14 DPI in WT mice, SPL metabolism may also play a modulatory role in the proliferative phase of wound healing [3,4,5,6,7]. By 28 DPI, the corneal wound should be in the remodeling phase of healing [3,4,5,6,7]. This could explain the absence of significant differences between WT and *SphK1^-/-^* SPL concentrations at 28 DPI.

Importantly, at 7 DPI, we observed significantly decreased concentrations of Cer, SM, and S1P in *SphK1^-/-^* mouse corneas compared to WT. In acute wounds, S1P has been shown to promote immune cell migration, increase vascular permeability, and inhibit PDGF-induced fibroblast proliferation [47,48,49]. S1P has also been reported to stimulate excessive production of collagens, which contributes to the formation of scar tissue [33,50,51]. Our previous study found that *SphK1^-/-^* mice had improved corneal wound healing and decreased neovascularization [34]. Perhaps these inflammatory and pro-fibrotic mechanisms of S1P and other major SPL species, which may be occurring around 7 DPI, are responsible for these differences in wound healing outcomes. The blunting of SPL concentrations, particularly at 1 and 14 DPI, and many significant differences in SPL concentrations at 7 DPI, may indicate that the first two weeks following a corneal wound are an important window of opportunity for the prevention of corneal scarring or angiogenesis [34].

The only effective treatment for vision loss due to corneal scarring is corneal transplantation, despite its various limitations and potential complications, including post-surgical fibrosis [52,53]. One of the underlying mechanisms in corneal fibrosis is the activation of stromal keratocytes into myofibroblasts during injury or disease. This process is critically influenced by cytokines, growth factors, and chemokines [5,6]. The SphK1-S1P axis also regulates myofibroblast differentiation, as observed in both SphK1 knockout studies and via inhibition of SphK1-S1P signaling [25,31,33,54,55]. Our group also reported that S1P signaling regulates both canonical and non-canonical pathways of TGF-β-mediated corneal fibrosis [32,33]. We observed that inhibition of SphK1 in human corneal fibroblasts induced reversal and prevention of fibrosis by inactivation of latent TGF-β signaling proteins, SphK1, SphK2, and S1PR3 [33]. Pharmacological inhibition of SphK1 has been reported to decrease inflammation, improve wound healing, and protect against fibrosis in many tissues [24,25,26]. *SphK1^-/-^* mice had decreased gene and protein expression of both fibrotic and inflammatory pathways in the liver [56]. SphK1 knockout also inhibited macrophage recruitment and polarization [56]. Reduced SphK1 activity and consequent decreases in S1P synthesis impair its angiogenic functions in skin wound healing [57]. Studies using S1PR antagonist FTY720 have observed protection against endoplasmic reticulum (ER) stress, mitochondrial dysfunction, fibrosis, and immune cell infiltration [26,54,58,59,60,61].

Overall, the SPL concentrations measured in this study are consistent with previous reports of SPL concentration in corneal tissues [34,62]. However, the observed levels of SPL concentration in uninjured corneas are not consistent with our previous study, where we observed increased concentration of SPLs in *SphK1^-/-^* mice corneas compared to WT [34]. One possible explanation for this may be differences in the collection of corneal tissue in the current study. As our previous study focused on neovascular invasion in the cornea, all corneal tissues sampled included the limbus [34]. The corneal limbus, comprised primarily of limbal epithelial stem cells, is a highly vascularized tissue that is critical to corneal epithelial wound healing [4,63]. Vascular concentrations of SPLs are significantly higher than those observed in the corneal tissue [34,38]. This is largely due to vascular endothelial cells, the function of which is known to be highly dependent on SPL metabolism [64,65]. Future studies comparing SPL concentrations in the corneal limbus, central cornea, layers of the cornea, and circulation are needed to understand how SPL metabolism shifts during wound healing.

## 5. Conclusions

In conclusion, our study demonstrates that SPL metabolism during corneal wound healing is modulated by SphK1, and by extension, S1P. It further demonstrates dramatic shifts in SPL concentrations throughout the stages of corneal wound healing in WT mice. Compared to these WT mice, *SphK1^-/-^* mice exhibited blunted corneal SPL metabolism throughout different stages of wound healing. These differences between genotypes are most noticeable within the first two weeks of corneal wound healing. Our findings highlight this timeframe as a window of opportunity for future therapeutic interventions that target SPL metabolism.

## Figures and Tables

**Table 1:** Expression of ceramide (Cer) species in wild-type (WT) and sphingosine kinase 1 knockout (*SphK1^-/-^*) mouse corneas after alkali burn. Within-genotype comparisons were conducted using paired *t*-tests comparing unburned with each timepoint, and between-genotype comparisons were evaluated using two-sample *t*-tests at each timepoint. Cer species are reported in pmol/mg. All data are represented as mean ± S.D. Significant differences within genotype are indicated as * *p* < 0.05; *** *p* < 0.001. Significant differences between genotypes are indicated as ^#^ *p* < 0.05; ^##^ *p* < 0.01. *n* = 6 for all groups. DPI: days post-injury.

	Uninjured	1 DPI	7 DPI	14 DPI	28 DPI	Uninjured	1 DPI	7 DPI	14 DPI	28 DPI
Cer Species	Wild-type					*SphK1^-/-^*				
C14:0	0.06 ± 0.02	0.05 ± 0.03	0.12 ± 0.05 *	0.05 ± 0.02	0.10 ± 0.03	0.05 ± 0.01	0.07 ± 0.04	0.06 ± 0.03 ^#^	0.04 ± 0.01	0.06 ± 0.03 ^#^
C16:0	5.92 ± 2.05	4.03 ± 1.29	10.25 ± 2.96 *	3.71 ± 1.81	8.42 ± 4.24	3.14 ± 0.56 ^#^	6.61 ± 3.73	4.98 ± 3.01 ^#^	3.33 ± 1.34	5.39 ± 2.31
C16 DH	0.11 ± 0.06	0.08 ± 0.03	0.19 ± 0.07	0.08 ± 0.05	0.16 ± 0.07	0.06 ± 0.03	0.14 ± 0.09	0.09 ± 0.04 ^#^	0.05 ± 0.02	0.10 ± 0.05
C18:1	0.19 ± 0.08	0.10 ± 0.04 *	0.29 ± 0.10	0.07 ± 0.27	0.22 ± 0.09	0.09 ± 0.02 ^#^	0.16 ± 0.09	0.14 ± 0.08 ^#^	0.10 ± 0.04	0.17 ± 0.07
C18:0	1.13 ± 0.05	0.56 ± 0.19 *	1.71 ± 0.57	0.11 ± 0.06	1.78 ± 0.84	0.53 ± 0.13 ^#^	1.04 ± 0.65	0.79 ± 0.39 ^##^	1.44 ± 1.60	1.09 ± 0.31
C20:0	1.33 ± 0.43	0.61 ± 0.19 *	1.16 ± 0.27	0.59 ± 0.20 *	1.17 ± 0.44	0.56 ± 0.09 ^##^	0.91 ± 0.52	0.50 ± 0.23 ^##^	0.73 ± 0.70	0.81 ± 0.31
C22:0	4.03 ± 1.29	2.97 ± 0.94	5.58 ± 1.58	2.22 ± 1.22	4.49 ± 2.08	2.07 ± 0.48 ^#^	4.59 ± 3.02	2.62 ± 1.48 ^##^	2.06 ± 1.31	2.96 ± 1.14
C24:1	8.44 ± 2.26	5.72 ± 2.27 *	12.30 ± 4.08	5.38 ± 3.66	10.34 ± 5.37	5.82 ± 1.57 ^#^	8.77 ± 5.79	5.75 ± 3.70 ^#^	3.93 ± 2.08	5.98 ± 3.42
C24:0	20.00 ± 4.80	12.49 ± 5.26 *	21.20 ± 6.00	9.60 ± 7.22 *	15.41 ± 6.61	11.27 ± 2.83	17.98 ± 13.08	9.84 ± 4.93 ^##^	5.62 ± 2.27 *	11.94 ± 7.39
C26:1	2.70 ± 0.65	2.55 ± 1.30	4.01 ± 1.31	1.79 ± 1.13	2.81 ± 1.11	1.49 ± 0.42 ^##^	3.07 ± 2.31	1.69 ± 0.85 ^##^	0.89 ± 0.25 *	1.61 ± 0.91
C26:0	16.47 ± 3.89	11.65 ± 6.41	8.31 ± 2.77 *	6.92 ± 7.06	8.58 ± 3.84 *	9.90 ± 2.58 ^##^	16.50 ± 13.94	4.63 ± 2.05 ***^,#^	2.85 ± 1.30 ***	8.37 ± 6.96

**Table 2:** Expression of monohexosylceramide (MHC) species in wild-type and sphingosine kinase 1 knockout (*SphK1^-/-^*) mouse corneas after alkali burn. Within-genotype comparisons were conducted using paired *t*-tests comparing unburned with each timepoint, and between-genotype comparisons were evaluated using two-sample *t*-tests at each timepoint. MHC species are reported in pmol/mg. All data are represented as mean ± S.D. Significant differences within genotype are indicated as * *p* < 0.05; ** *p* < 0.01; *** *p* < 0.001. Significant differences between genotypes are indicated as ^#^ *p* < 0.05; ^##^ *p* < 0.01. *n* = 6 for all groups. DPI: days post-injury.

	Uninjured	1 DP1	7 DPI	14 DPI	28 DPI	Uninjured	1 DPI	7 DPI	14 DPI	28 DPI
MHC Species	Wild-type					*SphK1^-/-^*				
C14:0	0.01 ± 0.00	0.01 ± 0.00	0.03 ± 0.01 *	0.01 ± 0.01	0.02 ± 0.02	0.00 ± 0.01	0.02 ± 0.01 **^,#^	0.02 ± 0.02	0.01 ± 0.01 *	0.01 ± 0.00 *
C16:0	1.20 ± 0.61	3.01 ± 0.96 *	4.05 ± 2.06 *	1.12 ± 0.98	3.03 ± 2.26	0.34 ± 0.17 ^#^	4.11 ± 1.26 ***	2.13 ± 2.34	3.37 ± 3.42	1.97 ± 1.18 *
C16 DH	0.03 ± 0.01	0.06 ± 0.03 *	0.09 ± 0.03 **	0.03 ± 0.02	0.06 ± 0.04	0.01 ± 0.00 ^#^	0.10 ± 0.03 **	0.05 ± 0.05	0.08 ± 0.07	0.05 ± 0.02 **
C18:1	0.06 ± 0.03	0.05 ± 0.02	0.09 ± 0.03	0.03 ± 0.03	0.07 ± 0.04	0.02 ± 0.01 ^#^	0.07 ± 0.02 **	0.05 ± 0.06	0.07 ± 0.05 *	0.05 ± 0.02 *
C18:0	1.01 ± 0.64	0.47 ± 0.23	1.40 ± 0.53	0.49 ± 0.29	1.62 ± 0.97	0.27 ± 0.11 ^#^	0.80 ± 0.33 *	0.83 ± 0.84	1.13 ± 1.01	1.06 ± 1.02
C20:0	1.46 ± 0.86	0.44 ± 0.10 *	0.94 ± 0.52	0.35 ± 0.13 *	1.38 ± 0.95	0.38 ± 0.16 ^#^	0.57 ± 0.19	0.61 ± 0.82	1.87 ± 3.20	1.60 ± 2.72
C22:0	6.16 ± 3.51	2.46 ± 0.94	3.69 ± 1.91	1.29 ± 0.67 *	3.88 ± 3.21	1.17 ± 0.59 ^#^	3.19 ± 1.32 **	2.19 ± 3.18	5.29 ± 8.84	3.43 ± 4.69
C24:1	1.83 ± 1.02	1.77 ± 0.58	3.05 ± 1.65	0.95 ± 0.70	2.27 ± 1.64	0.44 ± 0.22 ^#^	2.44 ± 1.22 **	1.67 ± 2.06	2.08 ± 2.01	1.16 ± 0.49 *
C24:0	6.62 ± 3.54	3.89 ± 1.65	6.10 ± 2.84	2.06 ± 0.97 *	5.18 ± 3.89	1.44 ± 0.64 ^#^	5.96 ± 2.82 **	3.39 ± 4.19	5.07 ± 5.85	3.37 ± 2.55
C26:1	0.18 ± 0.08	0.24 ± 0.08	0.18 ± 0.06	0.08 ± 0.02 *	0.14 ± 0.06	0.06 ± 0.02 ^#^	0.34 ± 0.15 **	0.10 ± 0.09	0.13 ± 0.09	0.09 ± 0.02 **
C26:0	0.72 ± 0.30	1.56 ± 0.54 *	0.65 ± 0.21	0.29 ± 0.12 **	0.37 ± 0.24	0.20 ± 0.10 ^##^	2.26 ± 1.16 **	0.48 ± 0.54	0.48 ± 0.37	0.28 ± 0.08

**Table 3:** Expression of sphingomyelin (SM) species in wild-type and sphingosine kinase 1 knockout (*SphK1^-/-^*) mouse corneas after alkali burn. Within-genotype comparisons were conducted using paired *t*-tests comparing unburned with each timepoint, and between-genotype comparisons were evaluated using two-sample *t*-tests at each timepoint. SM species are reported in pmol/mg. All data are represented as mean ± S.D. Significant differences within genotype are indicated as * *p* < 0.05; ** *p* < 0.01; *** *p* < 0.001. Significant differences between genotypes are indicated as ^#^ *p* < 0.05; ^##^ *p* < 0.01. *n* = 6 for all groups. DPI: days post-injury.

	Uninjured	1 DPI	7 DPI	14 DPI	28 DPI	Uninjured	1 DPI	7 DPI	14 DPI	28 DPI
SM Species	Wild-type					*SphK1^-/-^*				
C14:0	2.24 ± 0.57	1.47 ± 0.27 *	3.99 ± 0.58 ***	1.59 ± 0.59	2.48 ± 0.62	1.96 ± 0.60	2.24 ± 0.80	2.14 ± 1.41 ^#^	1.77 ± 0.66	2.04 ± 0.62
C16:0	78.71 ± 30.52	42.72 ± 24.84	129.09 ± 39.09	53.51 ± 33.34	71.23 ± 61.49	50.34 ± 9.34	67.02 ± 43.11	69.10 ± 46.42 ^#^	43.24 ± 14.76	72.28 ± 33.75
C16 DH	22.62 ± 5.65	18.05 ± 5.64	35.39 ± 9.30 *	14.80 ± 8.13	25.82 ± 8.90	15.19 ± 2.46 ^#^	22.66 ± 14.14	16.21 ± 7.96 ^##^	10.12 ± 2.50 *	18.59 ± 8.42
C18:1	0.87 ± 0.35	0.42 ± 0.18 *	2.29 ± 0.76 **	0.59 ± 0.27	2.47 ± 1.48 *	0.66 ± 0.17	0.57 ± 0.48	1.05 ± 0.58 ^#^	1.74 ± 1.61	1.65 ± 1.62
C18:0	45.43 ± 21.20	18.70 ± 4.47 *	52.31 ± 7.42	24.88 ± 7.31	59.15 ± 19.94	24.97 ± 3.93	29.60 ± 14.37	27.18 ± 16.03 ^##^	31.28 ± 15.83	34.16 ± 9.27 *^,#^
C20:0	24.52 ± 10.39	8.95 ± 1.98 *	21.01 ± 2.34	10.76 ± 3.50 *	23.20 ± 6.60	12.63 ± 2.48 ^#^	14.30 ± 6.86	11.14 ± 7.40 ^#^	18.22 ± 16.04	18.10 ± 11.35
C22:0	69.08 ± 22.11	36.67 ± 8.68 *	82.19 ± 4.97	33.79 ± 13.00 *	58.04 ± 12.68	40.78 ± 7.72 ^#^	48.99 ± 17.41	42.18 ± 26.30 ^#^	32.26 ± 17.32	44.49 ± 12.48 ^#^
C24:1	134.67± 32.67	72.22 ± 20.47 *	159.32 ± 20.97	79.53 ± 43.21	134.35 ± 29.88	88.49 ± 12.13 ^#^	106.60 ± 56.81	86.30 ± 54.67 ^#^	60.25 ± 20.60 **	84.37 ± 31.85 ^#^
C24:0	166.34 ± 48.64	86.31 ± 29.91 *	180.03 ± 29.42	90.24 ± 48.06 *	148.95 ± 31.25	101.60 ± 12.76 ^#^	128.62 ± 75.96	100.17 ± 60.06 ^#^	64.58 ± 16.73 **	109.31 ± 47.97
C26:1	36.97 ± 10.16	20.85 ± 7.11	68.21 ± 10.92 **	32.20 ± 6.20	70.51 ± 20.60 **	21.76 ± 3.96 ^#^	30.78 ± 17.66	38.13 ± 26.26 ^#^	29.60 ± 11.47	35.61 ± 7.74 **^,##^
C26:0	120.92 ± 34.69	55.62 ± 20.20 *	88.82 ± 15.73	58.97 ± 29.19 *	103.73 ± 22.45	72.10 ± 8.77 ^#^	84.09 ± 51.72	50.83 ± 30.72 ^#^	37.97 ± 8.76	69.23 ± 32.59

**Table 4:** Expression of ceramide-1-phosphate (C1P) species in wild-type and sphingosine kinase 1 knockout (*SphK1^-/-^*) mouse corneas after alkali burn. Within-genotype comparisons were conducted using paired *t*-tests comparing unburned with each timepoint, and between-genotype comparisons were evaluated using two-sample *t*-tests at each timepoint. C1P species are reported in pmol/mg. All data are represented as mean ± S.D. Significant differences between genotypes are indicated as ^#^ *p* < 0.05. *n* = 6 for all groups. DPI: days post-injury.

	Uninjured	1 DPI	7 DPI	14 DPI	28 DPI	Uninjured	1 DPI	7 DPI	14 DPI	28 DPI
C1P Species	Wild-type					*SphK1^-/-^*				
C14:0	0.03 ± 0.01	0.03 ± 0.02	0.13 ± 0.11	0.05 ± 0.04	0.10 ± 0.07	0.02 ± 0.01 ^#^	0.04 ± 0.03	0.06 ± 0.04	0.03 ± 0.01	0.07 ± 0.07
C16:0	0.09 ± 0.03	0.13 ± 0.07	0.32 ± 0.23	0.17 ± 0.12	0.24 ± 0.16	0.07 ± 0.01	0.14 ± 0.07	0.15 ± 0.13	0.09 ± 0.04	0.22 ± 0.20
C22:0	0.05 ± 0.03	0.06 ± 0.05	0.13 ± 0.09	0.07 ± 0.08	0.13 ± 0.08	0.03 ± 0.01	0.08 ± 0.06	0.07 ± 0.07	0.04 ± 0.02	0.10 ± 0.08
C24:1	0.09 ± 0.05	0.11 ± 0.08	0.25 ± 0.16	0.13 ± 0.11	0.28 ± 0.21	0.06 ± 0.01	0.12 ± 0.08	0.13 ± 0.14	0.06 ± 0.03	0.16 ± 0.13
C24:0	0.21 ± 0.08	0.26 ± 0.17	0.43 ± 0.42	0.23 ± 0.20	0.34 ± 0.22	0.14 ± 0.02	0.25 ± 0.16	0.20 ± 0.18	0.10 ± 0.06	0.23 ± 0.16

## Data Availability

Data supporting the findings are included within the paper and are available from the corresponding authors upon reasonable request.

## Supplementary Materials

The following supporting information can be downloaded at: https://www.mdpi.com/article/10.3390/cells15080733/s1. **Figure S1:** Expression of ceramide (Cer) species in wild-type (WT) and sphingosine kinase 1 (*SphK1^-/-^*) knockout mouse corneas after alkali burn. Each panel represents individual Cer species. (A) C14:0; (B) C16:0; (C) C:16 DH; (D) C:18:1; (E) C:18:0; (F) C20:0; (G) C22:0; (H) C24:1; (I) C24:0; (J) C26:1; (K) C:26:0. Within-genotype comparisons were conducted using paired *t*-tests comparing unburned with each timepoint, and between-genotype comparisons were evaluated using two-sample *t*-tests at each timepoint. Cer species are reported in pmol/mg. All data are represented as mean ± S.E.M. Significant differences within genotype indicated as * *p* < 0.05; *** *p* < 0.001. Significant differences between genotypes indicated as # *p* < 0.05; ## *p* < 0.01. *n* = 6 for all groups. DPI: Days post injury. **Figure S2:** Expression of monohexosylceramide (MHC) species in wild-type (WT) and sphingosine kinase 1 (*SphK1^-/-^*) knockout mouse corneas after alkali burn. Each panel represents individual MHC species. (A) C14:0; (B) C16:0; (C) C:16 DH; (D) C:18:1; (E) C:18:0; (F) C20:0; (G) C22:0; (H) C24:1; (I) C24:0; (J) C26:1; (K) C:26:0. Within-genotype comparisons were conducted using paired *t*-tests comparing unburned with each timepoint, and between-genotype comparisons were evaluated using two-sample *t*-tests at each timepoint. MHC species are reported in pmol/mg. All data are represented as mean ± S.E.M. Significant differences within genotype indicated as * *p* < 0.05; ** *p* < 0.01; *** *p* < 0.001. Significant differences between genotypes indicated as # *p* < 0.05; ## *p* < 0.01. *n* = 6 for all groups. DPI: Days post injury. **Figure S3:** Expression of sphingomyelin (SM) species in wild-type (WT) and sphingosine kinase 1 (*SphK1^-/-^*) knockout mouse corneas after alkali burn. Each panel represents individual SM species. (A) C14:0; (B) C16:0; (C) C:16 DH; (D) C:18:1; (E) C:18:0; (F) C20:0; (G) C22:0; (H) C24:1; (I) C24:0; (J) C26:1; (K) C:26:0. Within-genotype comparisons were conducted using paired *t*-tests comparing unburned with each timepoint, and between-genotype comparisons were evaluated using two-sample *t*-tests at each timepoint. SM species are reported in pmol/mg. All data are represented as mean ± S.E.M. Significant differences within genotype indicated as * *p* < 0.05; ** *p* < 0.01; *** *p* < 0.001. Significant differences between genotypes indicated as # *p* < 0.05; ## *p* < 0.01. *n* = 6 for all groups. DPI: Days post injury. **Figure S4:** Expression of ceramide-1-phosphate (C1P) species in wild-type (WT) and sphingosine kinase 1 (*SphK1^-/-^*) knockout mouse corneas after alkali burn. Each panel represents individual C1P species. (A) C14:0; (B) C16:0; (C) C22:0; (D) C24:1; (E) C24:0. Within-genotype comparisons were conducted using paired *t*-tests comparing unburned with each timepoint, and between-genotype comparisons were evaluated using two-sample *t*-tests at each timepoint. C1P species are reported in pmol/mg. All data are represented as mean ± S.E.M. Significant differences between genotypes indicated as # *p* < 0.05. *n* = 6 for all groups. DPI: Days post injury.
