# Supplementary material for: Sphingolipid Expression During Corneal Wound Healing in a Sphingosine Kinase 1 Knockout Model
Source: Cells. 2026 Apr 21;15(8):733. doi: 10.3390/cells15080733 (PMC13115462; doi:10.3390/cells15080733)
Supplement: Supplementary file 1 [file cells-15-00733-s001.zip › cells-4248415-supplementary.pdf]

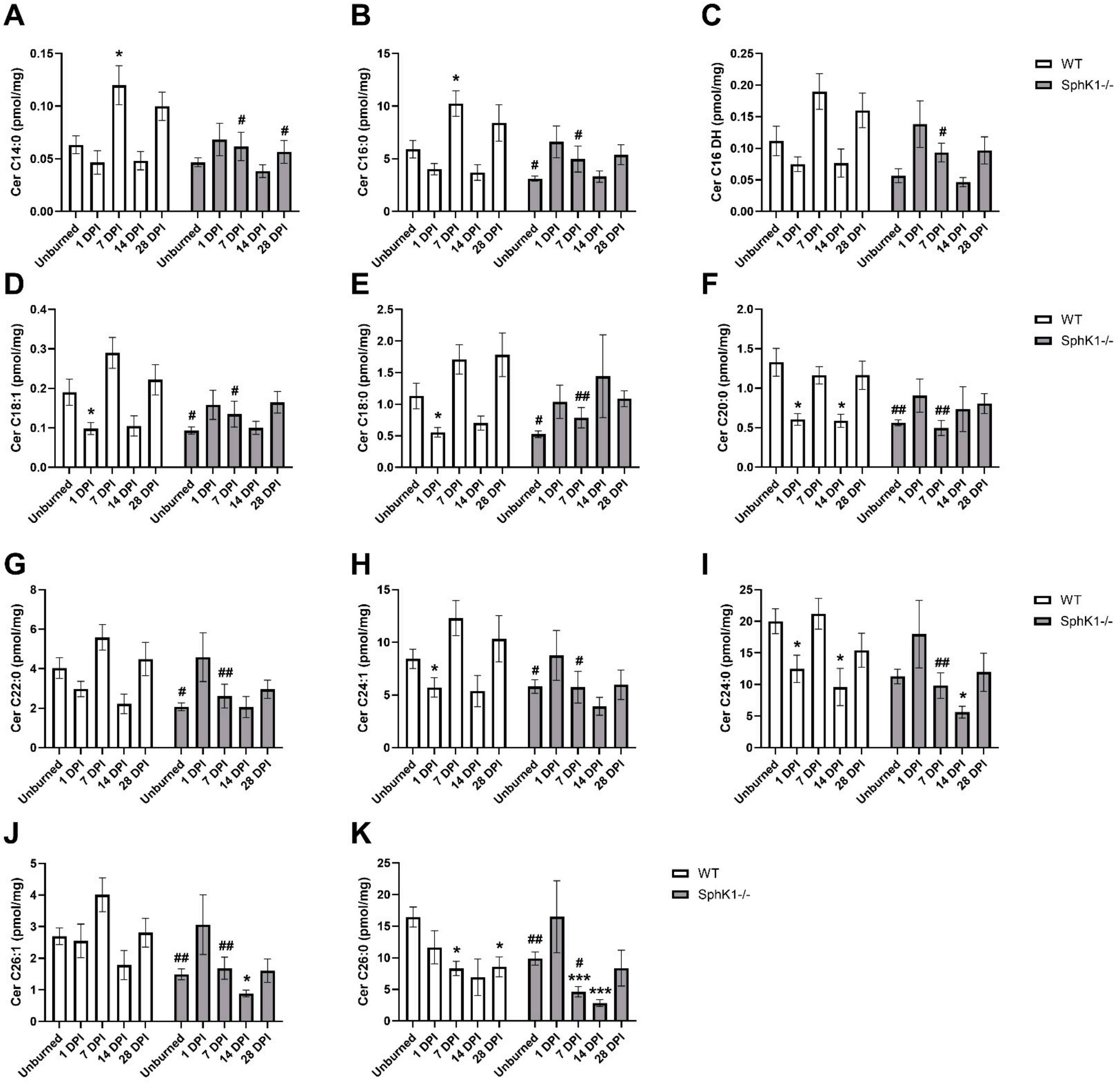

Figure S1: Expression of ceramide (Cer) species in wild-type (WT) and sphingosine kinase 1 (SphK1-/-) knockout mouse corneas after alkali burn. Each panel represents individual Cer species. A) C14:0; B) C16:0; C) C:16 DH; D) C:18:1; E) C:18:0; F) C20:0; G) C22:0; H) C24:1; I) C24:0; J) C26:1; K) C:26:0. Within-genotype comparisons were conducted using paired t-tests comparing unburned with each timepoint, and between-genotype comparisons were evaluated using two-sample t-tests at each

timepoint. Cer species are reported in pmol/mg. All data are represented as mean ± S.E.M. Significant differences within genotype indicated as *p<0.05; ***p<0.001. Significant differences between genotypes indicated as #p<0.05; ##p<0.01. n=6 for all groups. *DPI: Days post injury.*

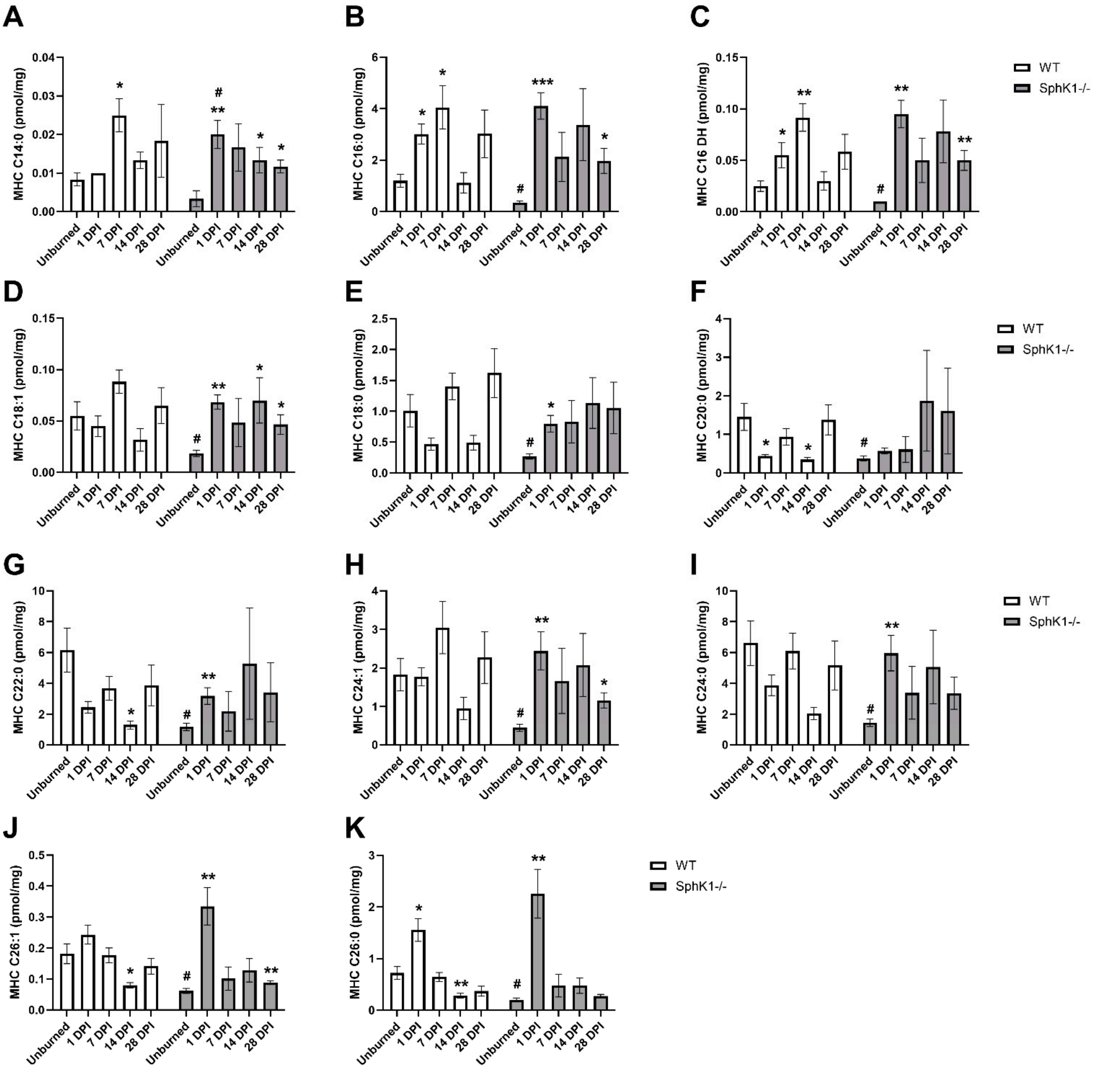

Figure S2: Expression of monohexosylceramide (MHC) species in wild-type (WT) and sphingosine kinase 1 (SphK1-/-) knockout mouse corneas after alkali burn. Each panel represents individual MHC species. A) C14:0; B) C16:0; C) C:16 DH; D) C:18:1; E) C:18:0; F) C20:0; G) C22:0; H) C24:1; I) C24:0; J)

C26:1; K) C:26:0. Within-genotype comparisons were conducted using paired t-tests comparing unburned with each timepoint, and between-genotype comparisons were evaluated using two-sample t-tests at each timepoint. MHC species are reported in pmol/mg. All data are represented as mean ± S.E.M. Significant differences within genotype indicated as *p<0.05; **p<0.01; ***p<0.001. Significant differences between genotypes indicated as #p<0.05; ##p<0.01. n=6 for all groups. *DPI: Days post injury.*

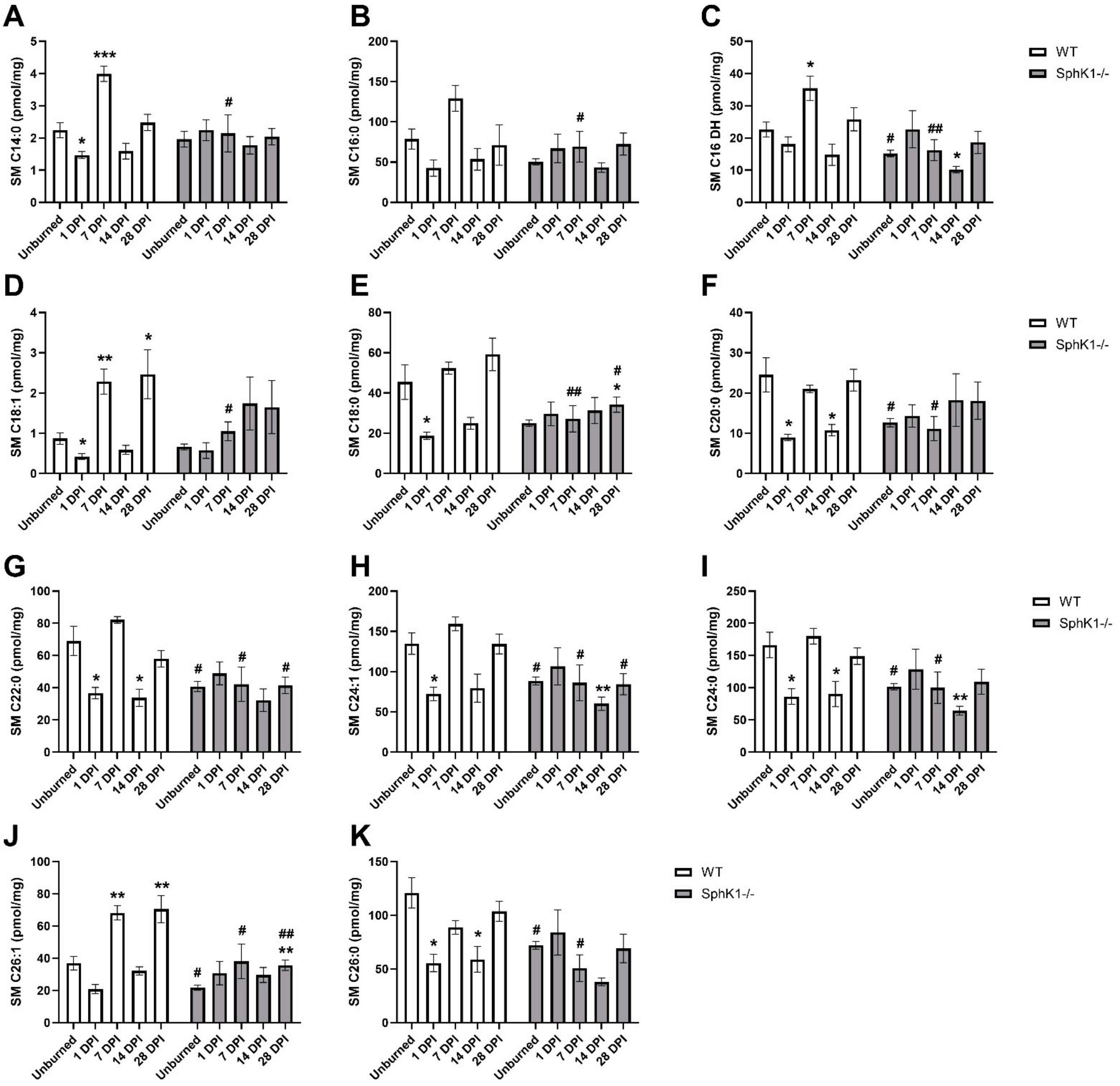

Figure S3: Expression of sphingomyelin (SM) species in wild-type (WT) and sphingosine kinase 1 (SphK1-/-) knockout mouse corneas after alkali burn. Each panel represents individual SM species. A) C14:0; B) C16:0; C) C:16 DH; D) C:18:1; E) C:18:0; F) C20:0; G) C22:0; H) C24:1; I) C24:0; J) C26:1; K) C:26:0. Within-genotype comparisons were conducted using paired t-tests comparing unburned with each timepoint, and between-genotype comparisons were evaluated using two-sample t-tests at each timepoint. SM species are reported in pmol/mg. All data are represented as mean ± S.E.M. Significant differences within genotype indicated as *p<0.05; **p<0.01; ***p<0.001. Significant differences between genotypes indicated as #p<0.05; ##p<0.01. n=6 for all groups. *DPI: Days post injury.*

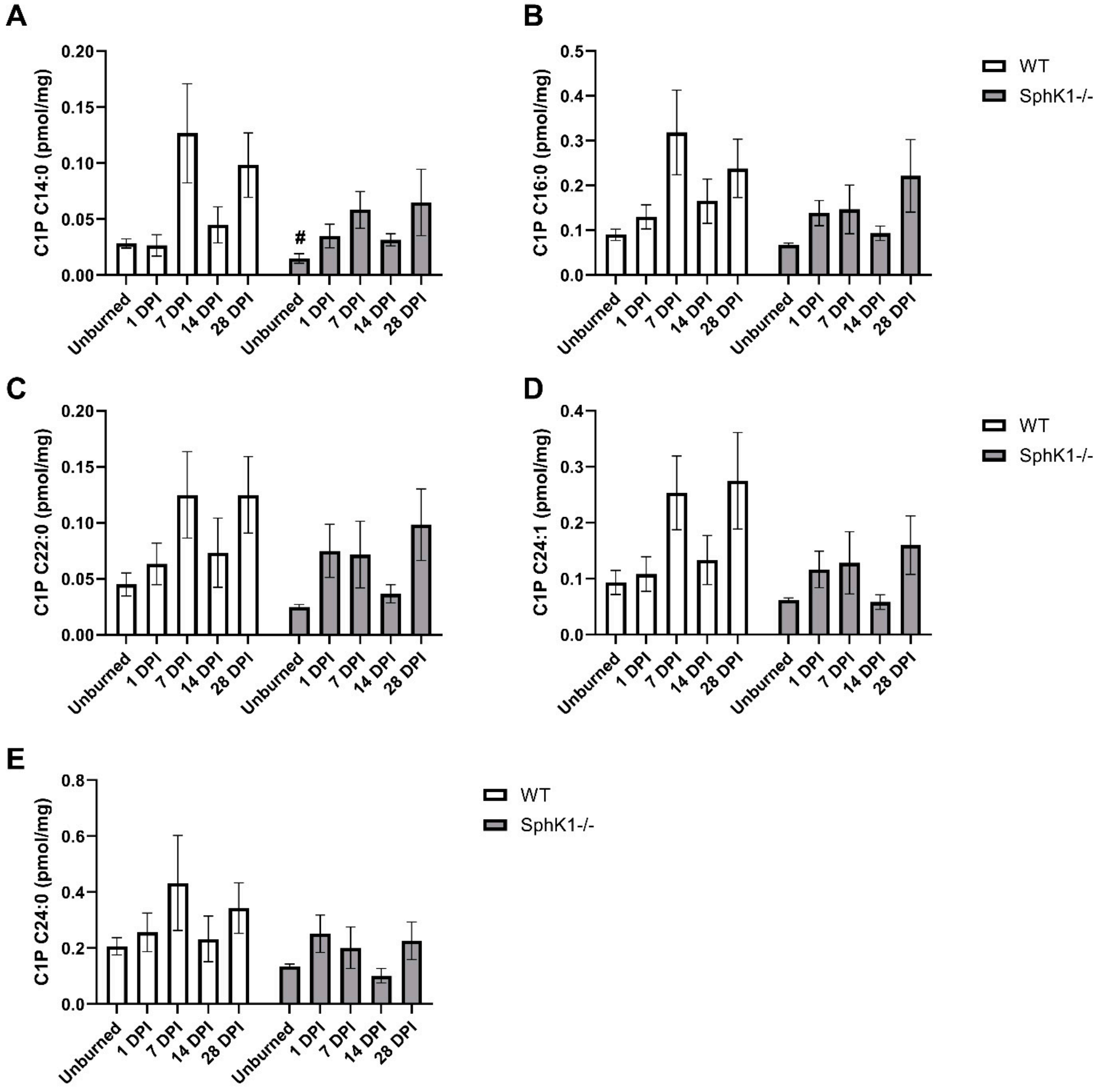

Figure S4: Expression of ceramide-1-phosphate (C1P) species in wild-type (WT) and sphingosine kinase 1 (SphK1-/-) knockout mouse corneas after alkali burn. Each panel represents individual C1P species. A) C14:0; B) C16:0; C) C22:0; D) C24:1; E) C24:0. Within-genotype comparisons were conducted using paired t-tests comparing unburned with each timepoint, and between-genotype comparisons were evaluated using two-sample t-tests at each timepoint. C1P species are reported in pmol/mg. All data are represented as mean ± S.E.M. Significant differences between genotypes indicated as #$p<0.05$. $n=6$ for all groups. *DPI: Days post injury.*
